# 7th Annual CANM IOM symposium Toronto, Ontario, Canada September 19-20, 2014

**Published:** 2014-10

**Authors:** Abeer S. Alsalamah

**Affiliations:** Department of Neurology, Prince Sultan Military Medical City, Riyadh, Kingdom of Saudi Arabia

## Abstract

The 7th Annual CANM IOM symposium was held in Toronto, Canada, on 19th-20th September 2014. The topics covered included intraoperative neurophysiological monitoring (IONM), anesthesia, brain mapping, spine surgery, EMG, EEG, and many others. The symposium provided a unique and interactive learning experience with case studies, internationally recognized speakers, and updates on the CANM Education Program.

***Lall RR, Lall RR, Hauptman JS, Munoz C, Cybulski GR, Koski T, Ganju A, Fessler RG, Smith ZA.***

Spine surgery carries an inherent risk of damage to critical neural structures. Intraoperative neurophysiological monitoring (IONM) is frequently used to improve the safety of spine surgery by providing real-time assessment of neural structures at risk. Evidence-based guidelines for safe and efficacious use of IONM are lacking and its use is largely driven by surgeon preference and medicolegal issues. Due to this lack of standardization, the preoperative sign-in serves as a critical opportunity for 3-way discussion between the neurosurgeon, anesthesiologist, and neuromonitoring team regarding the necessity for and goals of IONM in the ensuing case. This analysis contains a review of commonly used IONM modalities including somatosensory evoked potentials, motor evoked potentials, spontaneous or free-running electromyography, triggered electromyography, and combined multimodal IONM. For each modality the methodology, interpretation, and reported sensitivity and specificity for neurological injury are addressed. This is followed by a discussion of important IONM-related issues to include in the preoperative checklist, including anesthetic protocol, warning criteria for possible neurological injury, and consideration of what steps to take in response to a positive alarm. The authors conclude with a cost-effectiveness analysis of IONM, and offer recommendations for IONM use during various forms of spine surgery, including both complex spine and minimally invasive procedures, as well as lower-risk spinal operations.

*Lall RR, Lall RR, Hauptman JS, Munoz C, Cybulski GR, Koski T, Ganju A, Fessler RG, Smith ZA. Intraoperative neurophysiological monitoring in spine surgery: indications, efficacy, and role of the preoperative checklist. Neurosurg Focus 2012; 33: E10*.

