# Supplementary material for: 7th Annual CANM IOM symposium Toronto, Ontario, Canada September 19-20, 2014
Source: Neurosciences (Riyadh). 2014 Oct;19(4):343–9. (PMC4727680)
Supplement: Related texts

# Abstract: Augmentation of motor evoked potentials using multi-train transcranial electrical stimulation in intraoperative neurophysiologic monitoring during spinal surgery.

***Tsutsui S, Iwasaki H, Yamada H, Hashizume H, Minamide A, Nakagawa Y, Nishi H, Yoshida M.***

Transcranial motor evoked potentials (TcMEPs) are widely used to monitor motor function during spinal surgery. Improvements in transcranial stimulation techniques and general anesthesia have made it possible to record reliable and reproducible potentials. However, TcMEPs are much smaller in amplitude compared with compound muscle action potentials (CMAPs) evoked by maximal peripheral nerve stimulation. In this study, multi-train transcranial electrical stimulation (mt-TES) was introduced to enhance TcMEPs, and the optimal setting of mt-TES was investigated. In 30 patients undergoing surgical correction of spinal deformities (4 males and 26 females with normal motor status; age range 11-75 years), TcMEPs from the abductor hallucis (AH) and quadriceps femoris (QF) were analyzed. A multipulse (train) stimulus with an individual pulse width of 0.5 ms and an inter-pulse interval of 2 ms was delivered repeatedly (2-7 times) at different rates (2, 5, and 10 Hz). TcMEP amplitudes increased with the number of train stimuli for AH, with the strongest facilitation observed at 5 Hz. The response amplitude increased 6.1 times on average compared with single-train transcranial electrical stimulation (st-TES). This trend was also observed in the QF. No adverse events (e.g., seizures, cardiac arrhythmias, scalp burns, accidental injury resulting from patient movement) were observed in any patients. Although several facilitative techniques using central or peripheral stimuli, preceding transcranial electrical stimulation, have been recently employed to augment TcMEPs during surgery, responses are still much smaller than CMAPs. Changing from conventional st-TES to mt-TES has potential to greatly enhance TcMEP responses.

*Tsutsui S, Iwasaki H, Yamada H, Hashizume H, Minamide A, Nakagawa Y, Nishi H, Yoshida M. Augmentation of motor evoked potentials using multi-train transcranial electrical stimulation in intraoperative neurophysiologic monitoring during spinal surgery. J Clin Monit Comput 2014 Feb 16*.

# Abstract: Comparison of the Wake-up Test and Combined TES-MEP and CSEP Monitoring in Spinal Surgery.

***Chen B, Chen Y, Yang J, Xie D, Su H, Li F, Wan Y, Peng X, Zheng Z.***

**Study design:** A retrospective clinical analysis. **Objective:** To explore the effectiveness of the wake-up test and combined monitoring of transcranial electrical stimulation motor evoked potentials (TES-MEP) and cortical somatosensory evoked potentials (CSEP) in spinal surgery. **Summary of background data:** Currently, TES-MEP/CSEP combined monitoring has been increasingly recognized as the ideal approach to detect spinal neurophysiological compromise during spinal surgery but the merit of wake-up test is now in doubt. **Methods:** TES-MEP and CSEP combined monitoring were performed simultaneously in 426 patients who accepted spinal surgery in our department and wake-up tests were conducted in 23 patients due to positive neurophysiological monitoring results with uncertain causes, or continuing positive monitoring findings after all potential causes were resolved. Preoperative and postoperative neurological examination was performed as golden standard to detect irreversible spinal function compromise. All data were collected to compare the efficiency of MEP/CSEP combined monitoring and wake-up test. **Results:** Positive results of MEP/CSEP combined monitoring were recorded in 64 cases. Among them, the positive monitoring findings accorded with the results of neurological examination in 51 cases and the monitoring results didn’t match that of neurological examination in 13 cases. No false negative result was observed. The sensitivity of MEP/CSEP monitoring was 100%, the specificity was 96.5%, and the Youden index was 0.965. Wake-up tests were conducted in 23 cases in total. Eight patients who had positive monitoring findings completely matched the postoperative neurological examination results. While, in the other 15 cases with negative neurophysiological monitoring results, only 9 patients kept intact neurological function but the left 6 patients suffered compromised neurological function. The sensitivity of the wake-up test was 57.1%, the specificity was 100% and the Youden index was 0.571. **Conclusions:** Combined TES-MEP and CSEP monitoring, with its high sensitivity and specificity, is an effective method for monitoring spinal function during surgery and should be the preferred choice. The wake-up test is a useful complementary method to monitoring because of its high specificity.

*Chen B, Chen Y, Yang J, Xie D, Su H, Li F, Wan Y, Peng X, Zheng Z. Comparison of the Wake-up Test and Combined TES-MEP and CSEP Monitoring in Spinal Surgery. J Spinal Disord Tech 2013 Oct 30*.

# Abstract: Effects of partial neuromuscular blockade on facial nerve monitorization in otologic surgery.

***Kizilay A, Aladag I, Cokkeser Y, Miman MC, Ozturan O, Gulhas N.***

**Objective:** Neuromuscular blockade (NMB) is administered as part of a general anesthetic in order to keep the patient immobilized during surgery and has been known to hinder intraoperative neuromonitorization. The aim of this study was to determine the effects of different levels of NMB on electrical stimulation thresholds of the facial nerve during otologic surgery. **Material and methods:** Intraoperative facial nerve monitorization was performed in 29 patients with advanced middle ear disease. Electromyographic (EMG) responses were recorded by insertion of needle electrodes into the orbicularis oris and orbicularis oculi muscles. Minimal facial nerve stimulations causing EMG responses in the facial musculature were measured during full recovery from the effects of muscular relaxants and with 25%, 50%, 75% and 100% levels of NMB. These defined NMB levels were maintained by the administration of a drip infusion of atracurium and were assessed objectively by recording the hypothenar muscle action. **Results:** All of the patients had detectable EMG responses of the facial musculature at the 50% and 75% levels of NMB in response to the electrical stimulation of the facial nerve. The corresponding mean stimulation thresholds were 0.10 +/- 0.08 and 0.11 +/- 0.09 mA, respectively. No responses were measured in 31% of the patients when the level of peripheral NMB was 100%. **Conclusion:** This study suggests that a regulated 50% level of peripheral NMB provides reliable intraoperative EMG monitoring of the facial musculature in response to electrical stimulation and adequate anesthesia, with full immobilization of the patient.

*Kizilay A, Aladag I, Cokkeser Y, Miman MC, Ozturan O, Gulhas N. Effects of partial neuromuscular blockade on facial nerve monitorization in otologic surgery. Acta Otolaryngol 2003; 123: 321-324*.

# Abstract: Intraoperative neurophysiological monitoring in anterior lumbar interbody fusion surgery.

***Yaylali I, Ju H, Yoo J, Ching A, Hart R.***

**Purpose:** Somatosensory evoked potential (SSEP) and motor evoked potentials (MEP) are frequently fused to monitor neurological function during spinal deformity surgery. However, there are few studies regarding the utilization of intraoperative neuromonitoring during anterior lumbar interbody fusion (ALIF). This study presents the authors’ experience with intraoperative neuromonitoring in ALIF. **Methods:** A retrospective review of all patients undergoing ALIF with intraoperative neuromonitoring from November 2008 to July 2013 was performed. Factors including gender, operative time, blood loss, and number and levels of interbody fusions were analyzed as risk factors for interoperational alerts. **Results:** A total of 189 consecutive patients who underwent ALIFs were studied. All 189 patients had SSEP, and 131 patients had MEP as part of the intraoperative neuromonitoring in addition. The remaining 58 patients did not have MEP due to neuromuscular blockade requested by the exposure surgeon. There were no isolated intraoperative MEP changes. A total of 15 (7.9%) patients experienced intraoperative alerts. Thirteen (6.8%) of them were in SSEP. Two (1.1%) had MEP and SSEP changes together. None of these patients had new neurologic deficits postoperatively because of the surgeon’s responses to the intraoperative alert. Increased risk of SSEP changes was seen in patients undergoing fusion of both L4/5 and L5/S1 (P = 0.024) and longer surgical duration (P = 0.036). No correlation was found between age and positive SSEP changes (P > 0.05). **Conclusions:** Somatosensory evoked potential changes occur relatively, frequently, and intraoperatively during ALIF. No patients with positive intraoperative SSEP changes demonstrated new postoperational deficits. Concurrent fusion of both the L4/5 and L5/S1 levels was significant risk factors for SSEP changes leading to intraoperative alerts. Operative duration and increased blood loss during surgery trended toward but did not reach statistical significance.

*Yaylali I, Ju H, Yoo J, Ching A, Hart R. Intraoperative neurophysiological monitoring in anterior lumbar interbody fusion surgery. J Clin Neurophysiol 2014; 31: 352-355*.

# Abstract: Intraoperative motor evoked potential monitoring - a position statement by the American Society of Neurophysiological Monitoring.

***Macdonald DB, Skinner S, Shils J, Yingling C; American Society of Neurophysiological Monitoring.***

The following intraoperative MEP recommendations can be made on the basis of current evidence and expert opinion: (1) Acquisition and interpretation should be done by qualified personnel. (2) The methods are sufficiently safe using appropriate precautions. (3) MEPs are an established practice option for cortical and subcortical mapping and for monitoring during surgeries risking motor injury in the brain, brainstem, spinal cord or facial nerve. (4) Intravenous anesthesia usually consisting of propofol and opioid is optimal for muscle MEPs. (5) Interpretation should consider limitations and confounding factors. (6) D-wave warning criteria consider amplitude reduction having no confounding factor explanation: >50% for intramedullary spinal cord tumor surgery, and >30-40% for peri-Rolandic surgery. (7) Muscle MEP warning criteria are tailored to the type of surgery and based on deterioration clearly exceeding variability with no confounding factor explanation. Disappearance is always a major criterion. Marked amplitude reduction, acute threshold elevation or morphology simplification could be additional minor or moderate spinal cord monitoring criteria depending on the type of surgery and the program’s technique and experience. Major criteria for supratentorial, brainstem or facial nerve monitoring include >50% amplitude reduction when warranted by sufficient preceding response stability. Future advances could modify these recommendations.

*Macdonald DB, Skinner S, Shils J, Yingling C; American Society of Neurophysiological Monitoring. Intraoperative motor evoked potential monitoring - a position statement by the American Society of Neurophysiological Monitoring. Clin Neurophysiol 2013; 124: 2291-2316*.

# Abstract: Preoperative characteristics of auditory brainstem response in acoustic neuroma with useful hearing: importance as a preliminary investigation for intraoperative monitoring.

***Aihara N, Murakami S, Takahashi M, Yamada K.***

We classified the results of preoperative auditory brainstem response (ABR) in 121 patients with useful hearing and considered the utility of preoperative ABR as a preliminary assessment for intraoperative monitoring. Wave V was confirmed in 113 patients and was not confirmed in 8 patients. Intraoperative ABR could not detect wave V in these 8 patients. The 8 patients without wave V were classified into two groups (flat and wave I only), and the reason why wave V could not be detected may have differed between the groups. Because high-frequency hearing was impaired in flat patients, an alternative to click stimulation may be more effective. Monitoring cochlear nerve action potential (CNAP) may be useful because CNAP could be detected in 4 of 5 wave I only patients. Useful hearing was preserved after surgery in 1 patient in the flat group and 2 patients in wave I only group. Among patients with wave V, the mean interaural latency difference of wave V was 0.88 ms in Class A (n = 57) and 1.26 ms in Class B (n = 56). Because the latency of wave V is already prolonged before surgery, to estimate delay in wave V latency during surgery probably underestimates cochlear nerve damage. Recording intraoperative ABR is indispensable to avoid cochlear nerve damage and to provide information for surgical decisions. Confirming the condition of ABR before surgery helps to solve certain problems, such as choosing to monitor the interaural latency difference of wave V, CNAP, or alternative sound-evoked ABR.

*Aihara N, Murakami S, Takahashi M, Yamada K. Preoperative characteristics of auditory brainstem response in acoustic neuroma with useful hearing: importance as a preliminary investigation for intraoperative monitoring. Neurol Med Chir (Tokyo) 2014; 54: 267-271*.

# Abstract: Intraoperative neurophysiological monitoring of the spinal cord during spinal cord and spine surgery: a review focus on the corticospinal tracts.

***Deletis V, Sala F.***

Recent advances in technology and the refinement of neurophysiological methodologies are significantly changing intraoperative neurophysiological monitoring (IOM) of the spinal cord. This review will summarize the latest achievements in the monitoring of the spinal cord during spine and spinal cord surgeries. This overview is based on an extensive review of the literature and the authors’ personal experience. Landmark articles and neurophysiological techniques have been briefly reported to contextualize the development of new techniques. This background is extended to describe the methodological approach to intraoperatively elicit and record spinal D wave and muscle motor evoked potentials (muscle MEPs). The clinical application of spinal D wave and muscle MEP recordings is critically reviewed (especially in the field of Neurosurgery) and new developments such as mapping of the dorsal columns and the corticospinal tracts are presented. In the past decade, motor evoked potential recording following transcranial electrical stimulation has emerged as a reliable technique to intraoperatively assess the functional integrity of the motor pathways. Criteria based on the absence/presence of potentials, their morphology and threshold-related parameters have been proposed for muscle MEPs. While the debate remains open, it appears that different criteria may be applied for different procedures according to the expected surgery-related morbidity and the ultimate goal of the surgeon (e.g. total tumor removal versus complete absence of transitory or permanent neurological deficits). On the other hand, D wave changes--when recordable--have proven to be the strongest predictors of maintained corticospinal tract integrity (and therefore, of motor function/recovery). Combining the use of muscle MEPs with D wave recordings provides the most comprehensive approach for assessing the functional integrity of the spinal cord motor tracts during surgery for intramedullary spinal cord tumors. However, muscle MEPs may suffice to assess motor pathways during other spinal procedures and in cases where the pathophysiology of spinal cord injury is purely ischemic. Finally, while MEPs are now considered the gold standard for monitoring the motor pathways, SEPs continue to retain value as they provide specificity for assessing the integrity of the dorsal column. However, we believe SEPs should not be used exclusively--or as an alternative to motor evoked potentials--during spine surgery, but rather as a complementary method in combination with MEPs. For intramedullary spinal tumor resection, SEPs should not be used exclusively without MEPs.

*Deletis V, Sala F. Intraoperative neurophysiological monitoring of the spinal cord during spinal cord and spine surgery: a review focus on the corticospinal tracts. Clin Neurophysiol 2008; 119: 248-264*.

# Abstract: Spinal cord mapping as an adjunct for resection of intramedullary tumors: surgical technique with case illustrations.

***Quinones-Hinojosa A, Gulati M, Lyon R, Gupta N, Yingling C.***

**Objective:** Resection of intramedullary spinal cord tumors may result in transient or permanent neurological deficits. Intraoperative somatosensory evoked potentials (SSEPs) and motor evoked potentials are commonly used to limit complications. We used both antidromically elicited SSEPs for planning the myelotomy site and direct mapping of spinal cord tracts during tumor resection to reduce the risk of neurological deficits and increase the extent of tumor resection. **Methods:** In two patients, 3 and 12 years of age, with tumors of the thoracic and cervical spinal cord, respectively, antidromically elicited SSEPs were evoked by stimulation of the dorsal columns and were recorded with subdermal electrodes placed at the medial malleoli bilaterally. Intramedullary spinal cord mapping was performed by stimulating the resection cavity with a handheld Ojemann stimulator (Radionics, Burlington, MA). In addition to visual observation, subdermal needle electrodes inserted into the abductor pollicis brevis-flexor digiti minimi manus, tibialis anterior-gastrocnemius, and abductor halluces-abductor digiti minimi pedis muscles bilaterally recorded responses that identified motor pathways. **Results:** The midline of the spinal cord was anatomically identified by visualizing branches of the dorsal medullary vein penetrating the median sulcus. Antidromic responses were obtained by stimulation at 1-mm intervals on either side of the midline, and the region where no response was elicited was selected for the myelotomy. The anatomic and electrical midlines did not precisely overlap. Stimulation of abnormal tissue within the tumor did not elicit electromyographic activity. Approaching the periphery of the tumor, stimulation at 1 mA elicited an electromyographic response before normal spinal cord was visualized. Restimulation at lower currents by use of 0.25-mA increments identified the descending motor tracts adjacent to the tumor. After tumor resection, the tracts were restimulated to confirm functional integrity. Both patients were discharged within 2 weeks of surgery with minimal neurological deficits. **Conclusion:** Antidromically elicited SSEPs were important in determining the midline of a distorted cord for placement of the myelotomy incision. Mapping spinal cord motor tracts with direct spinal cord stimulation and electromyographic recording facilitated the extent of surgical resection.

*Quinones-Hinojosa A, Gulati M, Lyon R, Gupta N, Yingling C. Spinal cord mapping as an adjunct for resection of intramedullary tumors: surgical technique with case illustrations. Neurosurgery 2002; 51: 1199-1207*.

# Abstract: Topographic movie of intracranial ictal high-frequency oscillations with seizure semiology: epileptic network in Jacksonian seizures.

***Akiyama T, Chan DW, Go CY, Ochi A, Elliott IM, Donner EJ, Weiss SK, Snead OC 3rd, Rutka JT, Drake JM, Otsubo H.***

**Purpose:** We developed a technique to produce images of dynamic changes in ictal high-frequency oscillations (HFOs) >40 Hz recorded on subdural electroencephalography (EEG) that are time-locked to the ictal EEG and ictal semiology video. We applied this technique to Jacksonian seizures to demonstrate ictal HFO propagation along the homunculus in the primary sensory-motor cortex to visualize the underlying epileptic network. **Methods:** We analyzed intracranial ictal EEGs from two patients with intractable Jacksonian seizures who underwent epilepsy surgery. We calculated the degrees of increase in amplitude within 40-80, 80-200, and 200-300 Hz frequency bands compared to the interictal period and converted them into topographic movies projected onto the brain surface picture. We combined these data with the ictal EEGs and video of the patient demonstrating ictal semiology. **Key findings:** The ictal HFOs began in the sensory cortex and appeared concomitantly with the sensory aura. They then propagated to the motor cortex at the same time that focal motor symptoms evolved. As the seizure progressed, the ictal HFOs spread or reverberated in the rolandic region. However, even when the seizure became secondarily generalized, the ictal HFOs were confined to the rolandic region. In both cases, there was increased amplitude of higher frequency bands during seizure initiation compared to seizure progression. **Significance:** This combined movie showed the ictal HFO propagation corresponding to the ictal semiology in Jacksonian seizures and revealed the epileptic network involved in seizure initiation and progression. This method may advance understanding of neural network activities relating to clinical seizure generation and propagation.

*Akiyama T, Chan DW, Go CY, Ochi A, Elliott IM, Donner EJ, Weiss SK, Snead OC 3rd, Rutka JT, Drake JM, Otsubo H. Topographic movie of intracranial ictal high-frequency oscillations with seizure semiology: epileptic network in Jacksonian seizures. Epilepsia 2011; 52: 75-83*.

# Abstract: Low-threshold monopolar motor mapping for resection of lesions in motor eloquent areas in children and adolescents.

***Schucht P, Seidel K, Murek M, Stieglitz LH, Urwyler N, Wiest R, Steinlin M, Leibundgut K, Raabe A, Beck J.***

**Object:** Resection of lesions close to the primary motor cortex (M1) and the corticospinal tract (CST) is generally regarded as high-risk surgery due to reported rates of postoperative severe deficits of up to 50%. The authors’ objective was to determine the feasibility and safety of low-threshold motor mapping and its efficacy for increasing the extent of lesion resection in the proximity of M1 and the CST in children and adolescents. **Methods:** The authors analyzed 8 consecutive pediatric patients in whom they performed 9 resections for lesions within or close (≤ 10 mm) to M1 and/or the CST. Monopolar high-frequency motor mapping with train-of-five stimuli (pulse duration 500 µsec, interstimulus interval 4.0 msec, frequency 250 Hz) was used. The motor threshold was defined as the minimal stimulation intensity that elicited motor evoked potentials (MEPs) from target muscles (amplitude > 30 µV). Resection was performed toward M1 and the CST at sites negative to 1- to 3-mA high-frequency train-of-five stimulation. **Results:** The M1 was identified through high-frequency train-of-five via application of varying low intensities. The lowest motor thresholds after final resection ranged from 1 to 9 mA in 8 cases and up to 18 mA in 1 case, indicating proximity to motor neurons. Intraoperative electroencephalography documented an absence of seizures during all surgeries. Two transient neurological deficits were observed, but there were no permanent deficits. Postoperative imaging revealed complete resection in 8 patients and a very small remnant (< 0.175 cm3) in 1 patient. **Conclusions:** High-frequency train-of-five with a minimal threshold of 1-3 mA is a feasible and safe procedure for resections in the proximity of the CST. Thus, low-threshold motor mapping might help to expand the area for safe resection in pediatric patients with lesions located within the precentral gyrus and close to the CST, and may be regarded as a functional navigational tool. The additional use of continuous MEP monitoring serves as a safety feedback for the functional integrity of the CST, especially because the true excitability threshold in children is unknown.

*Schucht P, Seidel K, Murek M, Stieglitz LH, Urwyler N, Wiest R, Steinlin M, Leibundgut K, Raabe A, Beck J. Low-threshold monopolar motor mapping for resection of lesions in motor eloquent areas in children and adolescents. J Neurosurg Pediatr 2014; 13: 572-578*.
